# Fasting GLP-1 Levels and Albuminuria Are Negatively Associated in Patients with Type 2 Diabetes Mellitus

**DOI:** 10.3390/jpm14030280

**Published:** 2024-03-01

**Authors:** Cheol-Won Jang, Tae Yang Yu, Jin Woo Jeong, Se Eun Ha, Rajan Singh, Moon Young Lee, Seungil Ro

**Affiliations:** 1 Division of Endocrinology and Metabolism, Department of Medicine, School of Medicine, Wonkwang University, Iksan 54538, Republic of Korea; zero7370@naver.com (C.-W.J.); endocrinology1@wku.ac.kr (T.Y.Y.); jinu84@wkuh.org (J.W.J.); 2 Department of Physiology and Cell Biology, University of Nevada School of Medicine, Reno, NV 89557, USA; seeunh@med.unr.edu (S.E.H.); rajans@med.unr.edu (R.S.); 3 Department of Physiology, School of Medicine, Wonkwang University, Iksan 54538, Republic of Korea; lmy6774@wku.ac.kr; 4 RosVivo Therapeutics, Applied Research Facility, 1664 N. Virginia St., Reno, NV 89557, USA

**Keywords:** type 2 diabetes mellitus, glucagon-like peptide-1, albuminuria

## Abstract

Glucagon-like peptide-1 (GLP-1) is an incretin hormone known for its pivotal role in enhancing insulin secretion and reducing glucagon release from the pancreas. Diabetic nephropathy, which is characterized by albuminuria, represents a significant microvascular complication of diabetes. Most of the previous studies mainly focused on the therapeutic renal protective effect in clinical trials after the administration of GLP-1 receptor agonists (GLP-1 RAs), rather than before administration. Hence, this study aimed to investigate the association between fasting plasma GLP-1 levels and albuminuria before GLP-1 RA administration. A cross-sectional study was designed to evaluate the association between fasting plasma GLP-1 levels and albuminuria in patients with type 2 diabetes mellitus (T2DM). A cohort of 68 participants with T2DM was analyzed using data collected at Wonkwang University Hospital in Iksan, Korea. Logistic regression analysis was employed to determine the odds ratio (OR) and 95% confidence interval (CI) of the incidence of albuminuria between two groups categorized by fasting GLP-1 levels, low (Group L) and high GLP-1 (Group H). The OR (95% CI) for the incidence of albuminuria comparing Group L with Group H of fasting plasma GLP-1 levels was 3.41 (1.16–10.02), *p* = 0.03 after adjustment for relevant variables including age, gender, fasting plasma glucose, HbA1c, C-peptide, creatinine, and medication use [angiotensin-converting enzyme (ACE) inhibitors or angiotensin receptor blockers (ARBs), sodium-glucose cotransporter-2 (SGLT-2) inhibitors, and dipeptidyl peptidase-4 (DPP-4) inhibitors]. When analyzed as a continuous variable, each 1 pmol/L reduction in fasting plasma GLP-1 levels was associated with an OR (95% CI) of 1.67 (1.17–1.87), *p* = 0.02, following full adjustment. These results highlight a negative association between fasting plasma GLP-1 levels and the incidence of albuminuria in Korean patients with T2DM, before GLP-1 RA administration. These findings suggest that endogenous GLP-1 may have a beneficial impact in mitigating albuminuria.

## 1. Introduction

The prevalence of diabetes has continued to increase worldwide over the past decades. Similarly, in Korea, the prevalence of diabetic patients is 16.7% (19.2% in men and 14.3% in women) among adults over 30 years of age, with the total number of diabetic patients estimated to be 6.05 million, according to the Diabetes Fact Sheet in Korea 2021 [1].

Among the various microvascular complications of diabetes, diabetic nephropathy has emerged as the leading cause of end-stage renal disease. In Korea, the percentage of patients with diabetes-related end-stage renal disease cases increased from 19.5% in 1992 to 50.6% in 2012 [2]. Moreover, the total number of patients newly initiating renal replacement therapy for end-stage renal disease has drastically increased, rising from 10,000 in 2011 to 18,642 in 2019 in Korea [3]. Diabetic nephropathy carries significant clinical implications, serving as a main contributor to morbidity and mortality in patients with diabetes [4]. Additionally, it is known to increase the risk of cardiovascular disease and mortality even before reaching end-stage renal failure [5].

Albuminuria, defined as a urine albumin creatinine ratio (UACR) of 30 mg/g or higher, is observed in the early stages of renal disease associated with diabetes. The incidence of albuminuria, which contributes to both the diagnosis and prognosis of diabetic nephropathy, is well correlated with a decrease in glomerular filtration rate (GFR) and increased incidence of cardiovascular diseases [6,7,8]. 

The incretin hormone glucagon-like peptide-1 (GLP-1), which is comprised of 30 amino acids, is predominantly secreted by enteroendocrine L cells primarily found in the mucosa of both the small intestine and colon [9]. GLP-1 secretion exhibits a biphasic pattern in response to ingestion. An initial rapid surge in GLP-1 levels begins 15 to 30 min post-ingestion, followed by a second minor peak at 90 to 120 min [10]. The initial rapid reaction shortly following ingestion is associated with the proximal-distal loop regulated by acetylcholine and gastrin-releasing peptide [11]. The subsequent minor increase in GLP-1 occurs as ingested nutrients reach the intestinal lumen and directly stimulate distal L cells [12].

Although GLP-1 primarily stimulates pancreatic β-cells to enhance insulin secretion, it also induces a reduction in glucagon secretion. Additionally, GLP-1 increases glucose uptake and glycogen synthesis in peripheral tissues, delays gastric emptying, promotes satiety, thus suppressing appetite [13], and increases natriuresis and diuresis [14].

GLP-1 levels are decreased in patients with type 2 diabetes mellitus (T2DM) [15]. Since the approval of exenatide, the initial commercial GLP-1 receptor agonist (GLP-1 RA), by the US Food and Drug Administration in April 2005 for treating T2DM, GLP-1 RAs have become a crucial option for the treatment of T2DM. Apart from their glucose-lowering effects, this class of medication has been noted for not only providing cardiovascular protection but also demonstrating renal protective effects in previous cardiovascular clinical trials (CVOTs). Specifically, numerous GLP-1 RAs have shown improvements in albuminuria-based renal outcomes [16,17,18]. 

Nevertheless, most of the studies have focused on clinical trials examining the therapeutic effects of post-administration of GLP-1 RAs rather than pre-administration in patients with T2DM. Thus, this study aims to explore associations between fasting plasma GLP-1 levels and a major microvascular complication of diabetes, albuminuria, in patients with T2DM.

## 2. Methods

### 2.1. Study Population and Design

A cross-sectional study was designed to evaluate associations between fasting plasma GLP-1 levels and albuminuria on T2DM. Participants with T2DM were recruited from Wonkwang University Hospital in Iksan, Korea. The data on age, gender, smoking status, exercise frequency, blood pressure, body mass index (BMI), cholesterol level, renal function, diabetes status, insulin resistance, lipid-lowering drug use, antihypertensive drug use, anti-inflammatory marker, and albuminuria were collected and analyzed.

**Figure 1:**
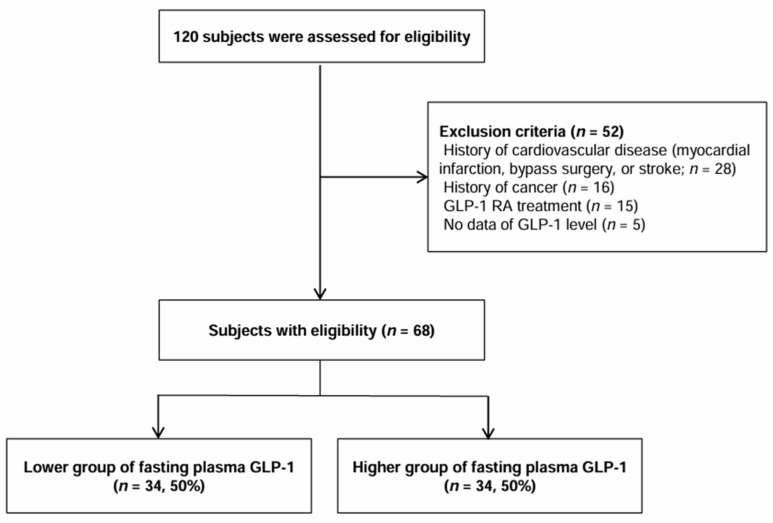
Selection of study population.

A total of 120 patients were recruited from August 2017 to May 2018. Among these participants, we excluded participants who were diagnosed with cardiovascular diseases (myocardial infarction, bypass surgery, or stroke, *n* = 28) or cancer (*n* = 16). In addition, we excluded those who received GLP-1 RA treatment (*n* = 15). Furthermore, those who had missing data (specifically, GLP-1 level, *n* = 5) were excluded. Several participants met more than two criteria. After these exclusions, the final study population included 68 participants (Figure 1). The study was conducted in accordance with the principles of the Declaration of Helsinki [19]. All study participants provided written informed consent prior to their enrollment in the study. The study protocol was approved by the institutional review board of Wonkwang University Hospital (WKUH 2017-07-025-001).

### 2.2. Measurement

Systolic and diastolic blood pressures were measured three times using a mercury sphygmomanometer. Each participant was seated and allowed to rest for at least 5 min before blood pressure was measured. The statistical analysis employed the average of these measurements. The heights and weights of participants were measured to the nearest 0.1 centimeter (cm) and 0.1 kilogram (kg), respectively. BMI was calculated by dividing the body weight in kg by the square of height in meters (m^2^). Waist circumference was measured at the level of the iliac crests, which usually represents the narrowest point of the torso. 

Venous blood samples were collected after overnight fasting. Plasma glucose, total cholesterol, high-density lipoprotein cholesterol (HDL), low-density lipoprotein cholesterol (LDL), and triglyceride levels were measured using a Hitachi Automatic Analyzer 7600 (Hitachi, Tokyo, Japan). Plasma active GLP-1 levels were measured by ELISA (immuno-Biological Laboratories Co., Ltd., Tokyo, Japan; Code No. 27784 GLP-1, Active form Assay Kit-IBL). Glycated hemoglobin (HbA1c) levels were measured using high-performance liquid chromatography. Serum high-sensitivity C-reactive protein (hs-CRP) levels were measured by immunoturbidimetry.

A random urine sample was obtained to measure creatinine and albumin levels. UACRs were calculated, and proteinuria was diagnosed as UACR > 30 mg/g. Estimated GFRs were calculated using the Modification of Diet in Renal Disease (MDRD) equation [20].

Insulin resistance was defined by the Homeostatic Model Assessment of Insulin Resistance (HOMA-IR), calculated as the product of fasting plasma insulin level (μU/mL) and fasting plasma glucose (FPG) level (mg/dL), divided by 405. β-cell insulin secretory function was evaluated by the Homeostatic Model Assessment of β-cell function [HOMA-β (%)], calculated as the product of 360 and fasting plasma insulin level (μU/mL), divided by glucose minus 63 [21].

T2DM was defined as a fasting glucose level of 126 mg/dL or higher, an HbA1c level of 6.5% or higher, self-reported physician’s diagnosis, or the use of antidiabetic medications. Hypertension was defined as an average systolic blood pressure of 140 mmHg or higher, a diastolic blood pressure of 90 mmHg or higher, self-reported physician’s diagnosis, or the use of antihypertensive agents.

### 2.3. Statistics Analysis

Continuous variables with normal distributions were presented as mean ± standard deviation, and continuous variables with non-normal distributions were presented as medians (interquartile range). Categorical data are expressed as frequencies and percentages. Student’s *t*-test or Mann–Whitney U test was used for comparisons of the characteristics of study participants at baseline. Pearson’s Chi-Square test was performed for categorical variables to compare baseline characteristics. According to fasting plasma GLP-1 levels, participants were divided into Group L (low, *n* = 34) and Group H (high, *n* = 34).

Logistic regression analysis was performed to evaluate the odds ratio (OR) and 95% confidence interval (CI) of incidence of albuminuria between Group L and Group H of fasting plasma GLP-1 levels and fasting plasma GLP-1 levels in reductions of 1 pmol/L as a continuous variable. In addition to the unadjusted model (Model 1), the analysis included covariates such as age, gender, metabolic parameters (fasting plasma glucose, HbA1c, and C-peptide), and a renal parameter (creatinine) (Model 2). Moreover, medications, known to potentially influence the study outcomes, including ACE inhibitors, ARBs, SGLT-2 inhibitors, and DPP-4 inhibitors were incorporated into the model (Model 3). The variance inflation factor (VIF) was calculated for the independent predictors. A VIF < 5 was considered optimal to warrant stability.

Two-sided probability values < 0.05 were considered to indicate statistical significance. All statistical analyses were performed using SPSS version 21 (IBM Co., Armonk, NY, USA) and R version 4.3.2 (R Foundation, Vienna, Austria; http://www.r-project.org/ (accessed on 10 November 2023)).

## 3. Results

We analyzed and characterized associations between GLP-1 levels and albuminuria in 68 participants [men, 63.2% (*n* = 43) and women, 36.8% (*n* = 25)] after exclusion of 52 patients among the 120 participants with T2DM (Figure 1). Table 1 summarizes the baseline clinical and biochemical characteristics of study participants with low and high plasma fasting GLP-1 levels (Group L and Group H). Participants in Group L had more men (76.5%, *n* = 26) compared to those in Group H (50.0%, *n* = 17, *p* = 0.04). Participants in Group L also had higher creatinine (0.9 ± 0.4 mL/min/1.73 m^2^) compared to those in Group H (0.7 ± 0.3 mL/min/1.73 m^2^, *p* = 0.04). In addition, participants in Group L had significantly higher UACR [55.0 (0.0–470.0)] compared to those in Group H [10.0 (0.0–60.0), *p* = 0.04]. Individuals in Group L exhibited a higher tendency for the presence of albuminuria (58.8%, *n* = 20) compared to those in Group H (32.4%, *n* = 11, *p* = 0.05). There were no significant differences in other parameters between the two groups.

Table 2 displays correlations between fasting plasma GLP-1 levels and metabolic, anthropometric, and biochemical parameters. In Pearson’s correlation analysis, the fasting plasma GLP-1 levels were positively correlated with estimated GFR but negatively correlated with UACR. In multiple regression analyses, the fasting plasma GLP-1 levels were negatively correlated with fasting plasma glucose and UACR.

Table 3 shows the ORs and 95% CIs for the incidence of albuminuria between Group L and Group H of fasting plasma GLP-1 levels and fasting plasma GLP-1 levels in reductions of 1 pmol/L as a continuous variable. In the unadjusted model (Model 1), the OR (95% CI) for the incidence of albuminuria comparing Group L with Group H of fasting plasma GLP-1 levels was 2.99 (1.11–8.05), *p* = 0.03. This association was maintained in Model 2, which is further adjusted for age, gender, fasting plasma glucose, HbA1c, C-peptide, and creatinine [OR (95% CI) = 3.12 (1.13–8.60), *p* = 0.03]. In Model 3, after further adjustment for the use of angiotensin-converting enzyme (ACE) inhibitors, angiotensin receptor blockers (ARBs), sodium-glucose cotransporter-2 (SGLT-2) inhibitors, and dipeptidyl peptidase-4 (DPP-4) inhibitors, the OR (95% CI) for the incidence of albuminuria comparing Group L with Group H of fasting plasma GLP-1 levels was 3.41 (1.16–10.02), *p* = 0.03.

As a continuous variable, fasting plasma GLP-1 levels were also negatively associated with the incidence of albuminuria. In Model 1, the OR (95% CI) for the incidence of albuminuria associated with a reduction of 1 pmol/L in fasting plasma GLP-1 levels was 1.56 (1.00–1.80) (*p* = 0.049). This negative association was apparent even after further adjusting for confounders in Model 2 [OR (95% CI) =1.62 (1.09–1.84), *p* = 0.03]. Finally, this inverse association was still significant after full adjustment for all confounding variables, as above [Model 3; OR (95% CI) = 1.67 (1.17–1.87), *p* = 0.02].

## 4. Discussion

In this study, we explored associations between fasting GLP-1 levels and albuminuria in patients with T2DM in Korea. We found that there is a negative association between plasma fasting GLP-1 levels and the incidence of albuminuria among patients with T2DM who did not receive GLP-1 RA medications. This negative association remained statistically significant after further adjustments for multiple associated confounders.

In addition to showing cardiovascular protective effects, previous randomized controlled studies demonstrated that GLP-1 RAs improve renal outcomes in patients with T2DM (16–18). A systematic review and meta-analysis of cardiovascular outcome trials including ELIXA (lixisenatide), LEADER (liraglutide), SUSTAIN-6 (semaglutide), EXSCEL (exenatide), HARMONY Outcomes (albiglutide), REWIND (dulaglutide), and PIONEER 6 (oral semaglutide), revealed that the use of GLP-1 RA resulted in a significant 17% reduction in a comprehensive kidney outcome [17,18,22,23,24,25,26,27,28,29]. This improved kidney outcome involved a decreased incidence of new-onset macroalbuminuria, the preservation of estimated GFR, slowed progression to end-stage renal disease, or a reduction in mortality attributable to kidney-related causes.

As the benefit of a decline in estimated GFR was not statistically significant and less notable compared to the impact observed with SGLT-2 inhibitors [30,31], this effect could mostly be attributed to a reduction in albuminuria. Moreover, GLP-1 RAs exhibit favorable outcomes [32] in reducing inflammation [33], managing obesity [34], protecting pulmonary function [35], and restoring gut microbiome composition [36].

While the precise mechanism remains uncertain, there are several hypotheses regarding the renal protective effect associated with the decrease in albuminuria, thought to be a class effect of GLP-1 RAs. The induction of natriuresis and diuresis by GLP-1 RAs has been proposed as a crucial underlying mechanism [14], involving the redistribution and reduction in Na^+^/H^+^ exchanger 3 (NHE3), predominantly located at the renal proximal tubules [37]. The reduced NHE3 activity may enhance sodium delivery to the macula densa, thereby influencing tubuloglomerular feedback, eventually resulting in renal afferent arteriolar vasoconstriction and reduced glomerular hyperfiltration [38]. Other hypotheses include the suppression of oxidative stress and local inflammation [39] via cyclic adenosine monophosphate (cAMP) and protein kinase A (PKA) mediated inhibition of renal NAD(P)H oxidase [40], the mitigation of oxidative stress-induced autophagy and endothelial dysfunction via downstream restoration of histone deacetylase 6 (HDAC6) facilitated by a GLP-1R-ERK1/2-dependent mechanism [41], and the reduction in circulating angiotensin II levels, which may induce natriuresis by the inhibiting renin-angiotensin system [42].

Importantly, the aforementioned studies have primarily focused on the therapeutic effects observed after the administration of GLP-1 RAs in T2DM patients, rather than assessing the conditions before administration in the patients. Thus, it is not yet clear how the role of endogenous GLP-1 in normal physiological conditions differs from those resulting from pharmacological GLP-1 RA treatment.

A previous study showed that postprandial GLP-1 levels were independently associated with microalbuminuria in newly diagnosed T2DM patients in China [43]. Patients with microalbuminuria demonstrated lower GLP-1 levels at 30 min and 120 min during a standard meal test compared to patients with normal albuminuria. In contrast, fasting plasma GLP-1 levels were not found to be associated with microalbuminuria (UACR: 30–299 mg/g). We included macroalbuminuria (UACR: >300 mg/g) in addition to microalbuminuria in T2DM patients and found that GLP-1 levels are indeed negatively associated with the full spectrum of albuminuria.

In addition to postprandial GLP-1 levels, fasting GLP-1 levels also bear significant clinical implications. Elevated fasting plasma GLP-1 concentrations have been correlated with decreased carbohydrate intake and reduced consumption of simple sugars, indicating a potential role of GLP-1 in the reward pathway regulating simple sugar intake [44]. Our study also suggests that elevated fasting plasma GLP-1 levels in patients with T2DM may be beneficial in reducing the incidence of albuminuria.

Next, we need to consider that other medications could affect the results of the study. Plasma GLP-1 levels could be increased by DPP-4 inhibitors, as they inhibit DPP-4 activity, which degrades GLP-1 in the peripheral circulation [45]. However, DPP-4 inhibitors exhibit a limited effect on increasing GLP-1 activity as GLP-1 has a minimal impact on insulin secretion by pancreatic β-cells in fasting conditions [46,47,48]. Thus, we selected to include the patients treated with DPP4-inhibitors in this study.

On the other hand, albuminuria could be reduced by ACE inhibitors and ARBs, which have been shown to delay progression to end-stage renal disease and reduce cardiovascular risk [49]. ACE inhibitors and ARBs decrease intraglomerular pressure by inhibiting angiotensin II-mediated efferent arteriolar vasoconstriction [50]. SGLT-2 inhibitors are anti-diabetic medications, which also reduce albuminuria [51]. SGLT-2 inhibitors decrease sodium reabsorption in the proximal convoluted tubules and increase sodium delivery to the macula densa cells, thereby reactivating tubuloglomerular feedback [52]. Consequently, SGLT-2 inhibitors inhibit the vasodilation of afferent arteriole, decrease intraglomerular pressure, and reduce albuminuria [53]. We accounted for the potential influence of medications on the results by considering them as confounding factors and provided the list of the medications used for the treatment of hypertension and diabetes in Supplementary Table S1.

Considering this is the era of personalized medicine, we need to raise the question of which candidates are suitable to benefit from exogenous GLP-1 RAs for not only glycemic control but also renal protection in T2DM patients. To the best of our knowledge, there has not been a large-scale randomized controlled trial (RCT) that has examined whether differences in basal GLP-1 levels resulted in different renal protection effects to GLP-1 RAs in T2DM patients. For future research endeavors, it becomes imperative to conduct meticulously designed longitudinal and large-scale studies to gain deeper insights into the impact of GLP-1 on albuminuria. 

It is important to acknowledge several limitations of this study. Firstly, this being a cross-sectional study, we should exercise caution in establishing causality between fasting plasma GLP-1 levels and the incidence of albuminuria. Secondly, the participants may not be representative of the entire Korean T2DM population, as they were self-selected, and the study was conducted using a sample from a single center. This introduces the possibility of selection bias, and the results may have limitations in terms of generalizability. Thirdly, the sample size in this study is comparatively limited. Conducting a larger randomized controlled study will serve to validate these results.

Despite these limitations, the study intentionally aimed to validate a direct negative association between plasma fasting GLP-1 levels and albuminuria. Its clinical implication lies in the direct quantification of plasma GLP-1 concentrations, bypassing the conventional post hoc validation of therapeutic effects following GLP-1 RA administration. Other strengths include measuring plasma GLP-1 as active GLP-1, not total GLP-1. Furthermore, we tried to adjust the use of medications that could affect plasma GLP-1 levels and albuminuria. These distinctive characteristics could contribute to the reliability and utility of this study.

## 5. Conclusions

In this study, we investigated the association between fasting GLP-1 levels and albuminuria in patients with T2DM in Korea. We observed a negative association between plasma fasting GLP-1 levels and the incidence of albuminuria among T2DM patients who were not administered GLP-1 RAs. This association persisted even after adjusting for confounding factors that might influence the results. These findings imply that endogenous GLP-1 may have a beneficial role in attenuating albuminuria.

## Figures and Tables

**Table 1:** Baseline characteristics of participants with type 2 diabetes mellitus in low (L) and high (H) groups of fasting plasma GLP-1 levels.

Variable	Fasting Plasma GLP-1 (pmol/L)			*p*-Value
	Group L (<1.83) *n* = 34	Group H (1.83–3.32) *n* = 34	Total *n* = 68	
Age (years)	59.3 ± 9.8	56.7 ± 10.0	58.8 ± 9.9	0.291
Men, *n* (%)	26 (76.5)	17 (50.0)	43 (63.2)	0.044
Systolic BP (mmHg)	131.3 ± 15.9	127.8 ± 11.9	129.5 ± 14.0	0.326
Diastolic BP (mmHg)	74.5 ± 11.2	76.4 ± 10.9	75.4 ± 11.0	0.493
Body mass index (kg/m^2^)	26.0 ± 3.4	25.2 ± 5.3	25.6 ± 4.4	0.505
Waist circumference (cm)	92.0 ± 8.6	93.0 ± 10.4	92.5 ± 9.4	0.754
Total cholesterol (mg/dL)	156.0 ± 31.3	169.6 ± 34.3	162.8 ± 33.3	0.093
HDL cholesterol (mg/dL)	46.9 ± 8.0	47.9 ± 10.7	47.4 ± 9.4	0.656
LDL cholesterol (mg/dL)	75.6 ± 17.7	82.7 ± 18.4	79.2 ± 18.3	0.109
Triglycerides (mg/dL)	148.5 (110.0–198.0)	160.0 (110.0–217.0)	155.0 (110.0–212.0)	0.840
Duration of diabetes (months)	121.9 ± 91.2	84.4 ± 79.4	102.9 ± 86.8	0.086
HbA1c (%)	8.3 ± 2.1	7.7 ± 1.5	8.0 ± 1.9	0.132
Fasting plasma glucose (mg/dL)	200.1 ± 78.5	177.0 ± 79.5	188.5 ± 79.3	0.231
Fasting insulin (uIU/mL)	12.9 (5.9–22.2)	20.0 (8.1–32.9)	14.5 (7.5–29.8)	0.130
HOMA-IR	5.4 (2.8–13.5)	10.9 (3.3–17.0)	6.7 (3.0–14.3)	0.275
HOMA-β (%)	27.4 (16.6–68.7)	41.9 (17.0–85.1)	32.7 (17.0–84.7)	0.448
Estimated GFR (mL/min/1.73 m^2^)	90.9 ± 27.9	103.6 ± 27.2	97.3 ± 28.1	0.079
Creatinine (mg/dL)	0.9 ± 0.4	0.7 ± 0.3	0.8 ± 0.3	0.040
hs-CRP (mg/dL)	0.4 (0.2–1.1)	0.9 (0.3–1.7)	0.5 (0.2–1.4)	0.067
GLP-1 (pmol/L)	1.4 ± 0.3	2.5 ± 0.5	2.0 ± 0.6	<0.001
UACR (mg/g)	55.0 (0.0–470.0)	10.0 (0.0–60.0)	10.0 (0.0–195.0)	0.036
Albuminuria, *n* (%)	20 (58.8)	11 (32.4)	31 (45.6)	0.051

Continuous variables with normal distributions are expressed as mean ± standard deviation, whereas continuous variables with non-normal distributions are expressed as median (interquartile range). Categorical variables are expressed as percentages (%). Abbreviations: GLP-1, glucagon-like peptide-1; BP, blood pressure; HDL, high density lipoprotein; LDL, low density lipoprotein; HOMA-IR, homeostatic model assessment for insulin resistance; HOMA-β, homeostasis model assessment of β-cell function; GFR, glomerular filtration rate; hs-CRP, high-sensitivity C-reactive protein; UACR, urine albumin creatinine ratio.

**Table 2:** Correlations between fasting plasma GLP-1 levels and metabolic, anthropometric, and biochemical parameters (Univariate and Multivariate Models).

**Univariate Model**	**Fasting Plasma GLP-1 (pmol/L)**		
	**r**	**r^2^**	***p*-Value**
Age (years)	−0.161	0.026	0.190
Men [*n* (%)]	0.154	0.024	0.210
Body mass index (kg/m^2^)	−0.039	0.002	0.749
Waist circumference (cm)	−0.051	0.003	0.740
Systolic BP (mmHg)	−0.173	0.030	0.172
Estimated GFR (ml/min/1.73 m^2^)	0.275	0.076	0.034
hs-CRP (mg/L)	−0.089	0.008	0.472
Total cholesterol (mg/dL)	0.126	0.016	0.307
Triglyceride (mg/dL)	0.099	0.010	0.422
LDL cholesterol (mg/dL)	0.075	0.006	0.543
HDL cholesterol (mg/dL)	−0.090	0.008	0.465
Fasting plasma glucose (mg/dL)	−0.060	0.004	0.626
Fasting plasma insulin (μU/mL)	0.162	0.026	0.197
HOMA-IR	0.109	0.012	0.388
HOMA- β (%)	−0.015	0.000	0.920
HbA1c (%)	−0.089	0.008	0.472
UACR (mg/g)	−0.252	0.064	0.038
**Multivariate Model**	**Fasting Plasma GLP-1 (pmol/L)**		
	**β**	**95% CI**	***p*** **-Value**
Fasting plasma glucose (mg/dL)	−0.002 ± 0.001	−0.004–0.000	0.047
UACR (mg/g)	−0.305 ± 0.133	−0.571–0.038	0.026

Abbreviations: GLP-1, glucagon-like peptide-1; BP, blood pressure; GFR, glomerular filtration rate; hs-CRP, high-sensitivity C-reactive protein; LDL, low density lipoprotein; HDL, high density lipoprotein; HOMA-IR, homeostatic model assessment for insulin resistance; HOMA-β, homeostasis model assessment of β-cell function; UACR, urine albumin creatinine ratio; CI, confidence interval.

**Table 3:** Odds ratios of fasting plasma GLP-1 levels in low (L) and high (H) groups and continuous GLP-1 levels in albuminuria.

	Fasting Plasma GLP-1 (pmol/L)				
	Group L (<1.83) *n* = 34	Group H * (1.83–3.32) *n* = 34	*p*-Value	Decline of 1 pmol/L as a Continuous Variable	*p*-Value
Model 1	2.987 (1.108–8.049)	1	0.031	1.556 (1.003–1.802)	0.049
Model 2	3.121 (1.133–8.603)	1	0.028	1.622 (1.091–1.843)	0.030
Model 3	3.410 (1.160–10.023)	1	0.026	1.673 (1.173–1.870)	0.018

Data are expressed as OR (95% CI). * 1 as a reference value. Model 1: unadjusted. Model 2: adjusted for age, gender, fasting plasma glucose, HbA1c, C-peptide, and creatinine. Model 3: adjusted for Model 2 plus the use of ACE inhibitors or ARBs, SGLT-2 inhibitors, and DPP-4 inhibitors.

## Data Availability

The data analyzed during the current study are available from the corresponding author upon request.

## Supplementary Materials

The following supporting information can be downloaded at: https://www.mdpi.com/article/10.3390/jpm14030280/s1, Table S1: A list of medications used by participants in low (L) and high (H) groups of fasting plasma GLP-1 levels.
