# Supplementary material for: Fasting GLP-1 Levels and Albuminuria Are Negatively Associated in Patients with Type 2 Diabetes Mellitus
Source: J Pers Med. 2024 Mar 1;14(3):280. doi: 10.3390/jpm14030280 (PMC10971117; doi:10.3390/jpm14030280)
Supplement: Supplementary file 1 [file jpm-14-00280-s001.zip › jpm-2872646-supplementary.pdf]

Journal of Personalized Medicine

MDPI

Supplementary Table S1. A list of medications used by participants in low (L) and high (H) groups of fasting plasma GLP-1 levels.

Medications	Fasting plasma GLP-1 (pmol/L)			p-value
	Group L* (<1.83) n = 34	Group H* (1.83-3.32) n = 34	Total* n = 68	
ACEi or ARB	16 (47.1)	14 (41.2)	30(44.1)	0.807
Beta blocker	9 (26.5)	4 (11.8)	13 (19.1)	0.217
Calcium channel blocker	10 (29.4)	7 (20.6)	17 (25.0)	0.575
Spironolactone	1 (2.9)	1 (2.9)	2 (2.9)	1.000
Hydralazine	0 (0.0)	1 (2.9)	1 (1.5)	1.000
Insulin	8 (23.5)	5 (14.7)	13 (19.1)	0.537
Metformin	29 (85.3)	32 (94.1)	61 (89.7)	0.425
Sulfonylurea	14 (41.2)	14 (41.2)	28 (41.2)	1.000
SGLT-2 inhibitor	2 (5.9)	9 (26.5)	11 (16.2)	0.048
Thiazolidinedione	7 (20.6)	3 (8.8)	10 (14.7)	0.304
DPP-4 inhibitor	21 (61.8)	21 (61.8)	42 (61.8)	1.000
Meglitinide	1 (2.9)	0 (0.0)	1 (1.5)	1.000
α-glucosidase inhibitor	1 (2.9)	0 (0.0)	1 (1.5)	1.000

*Data are expressed as n (%).
